# Delimitation and Phylogeny in *Fritillaria* Species (Liliaceae) Endemic to Alps

**DOI:** 10.3390/biology14070785

**Published:** 2025-06-28

**Authors:** Francesco Dovana, Lorenzo Peruzzi, Virgile Noble, Martino Adamo, Costantino Bonomi, Marco Mucciarelli

**Affiliations:** 1 Dipartimento di Bioscienze, Biotecnologie e Ambiente (DBBA), Campus Universitario “Ernesto Quagliariello”, Università Degli Studi di Bari “Aldo Moro”, Via Orabona 4, 70125 Bari, Italy; francesco.dovana@uniba.it; 2 Department of Biology, PLANTSEED Lab., University of Pisa, Via Derna 1, 56126 Pisa, Italy; lorenzo.peruzzi@unipi.it; 3 Conservatoire Botanique National Méditerranéen, 34 Avenue Gambetta, 83400 Hyères-les-Palmiers, France; v.noble@cbnmed.fr; 4 Department of Life Sciences and Systems Biology, University of Torino, Viale Pier Andrea Mattioli 25, 10125 Torino, Italy; martino.adamo@unito.it; 5 Botany Section, MUSE—Science Museum, Corso del Lavoro e della Scienza 3, 38121 Trento, Italy; costantino.bonomi@muse.it

**Keywords:** cpDNA, nrITS, endemic taxa, Maritime and Ligurian Alps, molecular phylogeny, taxonomy

## Abstract

The number of *Fritillaria* species native to the Alps has long been debated, and observational biases due to the short flowering periods and the scattered distributions of endemic *Fritillaria* populations along the mountain range have probably made the task of botanists more complicated. Moreover, previous phylogenetic studies in *Fritillaria* have considered alpine taxa only marginally. To test species boundaries within the *F. tubaeformis* species complex and to study their phylogenetic relationships, intra- and inter-specific genetic variability of sixteen samples belonging to four *Fritillaria* species was carried out in different localities of the Maritime and Ligurian Alps, with extensions to the rest of the Alpine arc. The combined use of five plastid DNA markers (*mat*K, *ndh*F, *rpl*16, *rpo*C1, and *pet*A-*psb*J) and nrITS showed that *F. tubaeformis* and *F. burnatii* are phylogenetically independent taxa, fully confirming morphological and morphometric divergences and, that *F. burnatii* is not related phylogenetically to the central European *F. meleagris*. Our phylogenetic study also supports the separation of *F. tubaeformis* from *F. moggridgei*, pointing to environment/ecological constraints or reproductive barriers as possible causes of their distinct evolutionary status. Our analysis also showed that the mountain endemic *F. involucrata* is not closely related to *F. tubaeformis*, contrasting with previous studies. The phylogenetic analysis of the nrITS region supports a close relationship between *F. burnatii* and *F. moggridgei*, but with low statistical support.

## Simple Summary

The Alpine Arc has acted as a diversification centre for many taxa of plants, and today it hosts an impressively rich biodiversity. However, the number of genera or subgeneric lineages containing species endemic to the Alps is very low. The reasons for such few and species-poor local diversification events are to be found in Quaternary climatic oscillations, and particularly glaciations, which prevented the evolution of species-rich lineages. The genus *Fritillaria* includes more than 140 species worldwide, but only a few are endemic to the Alps. A correct delimitation of the different species distributed on the Alps would help to assign correct taxonomic ranks to these endemics when their DNA is used in phylogenies based on a large dataset. This study clarifies the taxonomic status of *Fritillaria* taxa endemic to the Alps, only marginally studied so far on phylogenetic grounds. Based on our results, we recognise *F. tubaeformis*, *F. moggridgei*, and *F. burnatii* at species rank.

## 1. Introduction

The Alps are the highest mountains in Europe and are centres of diversity and endemism for many species [1,2,3]. The large climatic oscillations and physiographic heterogeneity typical of the alpine habitats have been key drivers of diversification and speciation in many alpine plants [3,4]. Located at the southern edge of the alpine arc, the Maritime and Ligurian Alps lie at the intersection of temperate and Mediterranean climates, making them particularly important for the study of plant evolution. These areas acted as key refugia during the last glacial maximum [5], sheltering numerous endemic species [6]. During Pleistocene glaciations, many species were pushed toward ice-free peripheral ranges, with the south-western and southern Alps serving as major refugia [7,8].

Here, we focus on the genus *Fritillaria*. It is the largest genus of the tribe Lilieae (Liliaceae) and includes about 140 bulbous perennial species occurring over a wide range of climates and habitats in the temperate regions of the Northern Hemisphere [9,10]. The Mediterranean area is generally considered the modern abundance centre of *Fritillaria* [11] and more specifically, a secondary diversification area of species distributed across Europe (mainly Greece), the Middle East, and North Africa (*F.* subg. *Fritillaria*, clade A *sensu* [12,13]). During the Late Pliocene, the onset of a seasonal climate in the Mediterranean Basin [2,14] promoted species diversification in this group of plants and several *Fritillaria* species further dispersed into Europe [12,13,15].

However, when we look at the number of *Fritillaria* species endemic to the Alps, we find a situation significantly different from the rest of the Mediterranean basin. Although hosting one of the most species-rich floras of the world, the Alps witnessed a rather limited number of local diversification events. This has resulted in a rather low number of endemic lineages compared to the overall number of endemic species in this mountain range [16]. It has been estimated that no more than 9% of alpine endemics have originated or diversified in situ (species diversification *sensu* Kadereit [16]). In this geographical context, the genus *Fritillaria* is exemplary of this paradigm, since only four species are endemic to the Alps.

Within the Alpine boundary, *Fritillaria involucrata* All. is endemic to south-eastern France and north-western Italy. It can be found on the Prealps, Maritime Alps [17,18,19], and Ligurian Alps [19,20,21,22]. This species is found on fresh and moderately fertile soils, in rocky open woods or fringes between 400 and 1500 m a.s.l. [19]. In the Ligurian Alps, it grows in open woods of *Ostrya carpinifolia* Scop. and in woodlands of beech forests or in clearings on calcareous soils (U. Ferrando, *pers. comm.*). According to the phylogenetic analysis published by Day et al. [12], which was based on a combined plastid dataset, *F. involucrata* would be paraphyletic with respect to *F. tubaeformis*. No further information is currently available on the phylogenetic position of this very rare species.

In the literature, taxa of the *F. tubaeformis* complex have been variously treated (see Mucciarelli et al. [23] for details): a single variable species with no infraspecific taxa [24,25,26], a single species with two infraspecific taxa, *F. tubaeformis* Gren. & Godr. subsp. *tubaeformis* and *F. tubaeformis* subsp. *moggridgei* (Boiss. & Reut. ex Planch.) Rix [27,28], three distinct taxa belonging to two different species: *F. tubaeformis* subsp. *tubaeformis*, *F. tubaeformis* subsp. *moggridgei, F. meleagris* subsp. *burnatii* (Planch.) Rix for Rix [29,30], or more recently as *F. tubaeformis* subsp. *tubaeformis*, *F. tubaeformis* subsp. *moggridgei* and *F. burnatii* (Planch.) Backh. [18,19]. Finally, the three taxa were treated as different species by Tison and de Foucault [31]: *F. tubaeformis* Gren. and Godr., *F. moggridgei* (Boiss. & Reut. ex Planch.) L.A.Cusin and *F. burnatii* (Planch.) Backh.

In line with the results presented in this study, we adopted the nomenclatural approach proposed by Tison and de Foucault [31] who recognised the species status for the three taxa of the *F. tubaeformis* complex. In accordance with the previous literature based on a morphological approach [19,31], Mucciarelli et al. [23] successfully distinguished the three taxa on morphometric grounds. In particular, the perigone is sub-rectangular in both *F. tubaeformis* and *F. moggridgei*, while it is typically rounded (U-shaped) in *F. burnatii* [23]. However, a comprehensive phylogenetic appraisal of this complex of species is presently lacking.

Most analyses to date have focused mainly on the genetic differentiation among the populations of *F. moggridgei* and *F. burnatii* [32,33], leaving their phylogenetic relationships—including those with respect to *F. tubaeformis* s. str.—unresolved.

To further test species boundaries within the three taxa, an intra- and inter-specific geographically oriented sampling was carried out, according to the most recent taxonomic and biogeographic revisions [23,34]. A multilocus dataset, combining plastid markers (including *mat*K, *ndh*F, *rpl*16, *rpo*C1, and *pet*A-*psb*J) with the nuclear ITS region, was used to assess variability and illustrate phylogenetic relationships among alpine *Fritillaria* taxa.

The most recent taxonomic hypothesis to be tested sees *F. tubaeformis* and *F. burnatii* as genetically other than morphologically independent taxa, and the former species further split into two subspecies [23]. A plastid and nuclear DNA phylogenetic approach was applied to examine:

(a) The phylogenetic relationships between *F. tubaeformis* and *F. burnatii*.
(b) The phylogenetic placement of *F. tubaeformis* and *F. burnatii* with respect to the central European *F. meleagris* and to the SW Alpine endemic *F. involucrata*.
(c) The taxonomic status of the two putative subspecies within *F. tubaeformis*.

## 2. Materials and Methods

### 2.1. DNA Extraction and PCR Amplification

Genomic DNA extraction was performed following two protocols [32,35]. The *mat*K partial region, *rpl*16 intron, *rpo*C1 intron, *pet*A-*psb*J intergenic spacer were amplified using primers and conditions summarised by Mucciarelli and Fay [32]. For *mat*K partial region in addiction BF and CR primers [36] were used. The *ndh*F region was amplified with primers 1318 and 2110 [37] following the conditions summarised by Marques et al. [38]. The ITS region was amplified using the primers ITS1/ITS4 or ITS-p5/ITS-u4 following the protocol provided by Cheng et al., 2016 [39].

### 2.2. Species Distribution Ranges in the F. tubaeformis Species Complex

In order to show the distribution of *F. burnatii*, *F. moggridgei*, and *F. tubaeformis,* distribution maps were produced using ConR package implemented in R package (version 4.1.1) [40]. Occurrence data were retrieved using online resources [41,42,43].

### 2.3. Sequence Alignment, Dataset Assembly, and Phylogenetic Analysis

Sequences were assembled and edited in Geneious v. 11.1.5 [44] and then submitted to GenBank^®^ (www.ncbi.nlm.nih.gov/genbank/ (accessed on 25 March 2023)). In cpDNA analysis, a total of eighteen accessions of *Fritillaria* were investigated in this work, while two accessions of *Lilium* from GenBank were used as outgroups. Table 1, Table 2 and Table 3 list accession names, DNA/herbarium voucher codes, and sequences used in the phylogenetic analysis, together with information on their origin. The accessions investigated in this study accounted for 74 new sequences belonging to five different taxa. Four taxa were sampled in the alpine region according to our previous studies [23,34]. Sequences of *Lilium superbum* and *Lilium bakerianum* were chosen as the outgroup taxa. Sequences of *mat*K region, *ndh*F, *rpl*16 intron, *rpo*C1 intron, *pet*A-*psb*J intergenic spacer, and nrITS region were separately aligned with MAFFT v. 7.017 [45] and then manually adjusted using Geneious v. R 11.1.4. For all independent datasets, five separate maximum likelihood (ML) analyses were run using W-IQ-TREE (version 1.6.12) [46]. The SH-like approximate likelihood ratio test (with 1000 replicates) and ultrafast bootstrap approximation (UFB) (1000 replicates) [47] were used to evaluate the reliability of clades (data available in Supplementary). No topological conflict was detected in the phylogenetic analyses of the *mat*K and *ndh*F regions, *rpl*16 and *rpo*C1 introns, and *pet*A-*psb*J intergenic spacer datasets (Figure S1a–e), so a combined dataset was created by concatenating the *mat*K, *ndh*F, *rpl*16, *rpo*C1, and *pet*A-*psb*J alignments using Geneious v. R 11.1.4.

For the analysis of the nrITS region, a dataset was used consisting of nine new sequences generated in this study: three of *F. burnatii*, one of *F. involucrata*, three of *F. moggridgei* and two of *F. tubaeformis*. Additionally, two sequences from *F. meleagris* were included, along with *Lilium bakerianum* and *Lilium superbum*, which were used as outgroups (Table 3). Partition Finder 2 [48] was used to estimate the best partitioning schemes and evolution models for each subset with MrBayes option. The concatenate cpDNA and nrITS datasets were analysed using Bayesian inference (BI) and maximum likelihood (ML) criteria. BI was performed with MrBayes v.3.2 [49] with four incrementally heated simultaneous Monte Carlo Markov Chains (MCMC) run for 10 million generations for cpDNA and nrITS alignment. Trees were sampled every 1000 generations, and the first 2500 trees were discarded as “burn-in” (25%). For the remaining trees, a majority rule consensus tree showing all compatible partitions was computed to obtain estimates for Bayesian Posterior Probabilities (BPP). The ML for the concatenated dataset was performed with IQ-TREE 2 [50]. The best models were selected using ModelFinder implemented in IQ-TREE 2 [51].

**Table 2:** *Fritillaria* samples used in cpDNA phylogenetic analyses together with GenBank accession numbers.

Taxa	References	*mat*K	*ndh*F	*rpl*16	*rpo*C1	*pet*A-*psb*J
*F. burnatii* (TEN1-20p2017)	this study	MN917987	MN917996	MN918014	-	MN918005
*F. burnatii* (TEN2-04p2013)	this study	-	MN917995	MN918015	-	MN918004
*F. burnatii* (SER-05p2013)	this study	MN917986	-	MN918013	MN918023	MN918003
*F. burnatii* (TN01)	this study	-	OP021726	-	OP021738	OP021733
*F. burnatii* (TN02)	this study	-	OP021727	-	OP021739	OP021732
*F. burnatii* (TN03)	this study	-	OP021728	-	OP021740	OP021734
*F. burnatii* (TN04)	this study	-	OP021729	-	OP021741	OP021735
*F. involucrata* (MEN-14p2016)	this study	MN917988	MN917997	MN918016	-	-
*F. involucrata* (HYE-CB976)	this study	-	OP021730	OP021736	OP021742	-
*F. meleagris* (GER-11p2015)	this study	MN917989	MN917998	MN918020	MN918025	MN918008
*F. meleagris* (Chase 2566 (K), Kew 1990-3088)	[11]	AY624445		AY624393		
*F. moggridgei* (MAR-07p2014)	this study	MN917992	MN917999	PV832077	-	MN918012
*F. moggridgei* (CRA-12p2014)	this study	MN917990	PV832078	MN918017	-	MN918009
*F. moggridgei* (FRO-00p2013)	this study	MN917991	-	MN918021	MN918027	MN918010
*F. moggridgei* (PLU-08p2014)	this study	PV832076	MN918000	MN918022	MN918026	MN918011
*F. moggridgei* (HYE-CB6181)	this study	-	OP021731	OP021737	-	-
*F. tubaeformis* (GLE-10p2013)	this study	MN917993	MN918001	MN918018	MN918028	MN918006
*F. tubaeformis* (GLE-09p2015)	this study	MN917994	MN918002	MN918019	MN918024	MN918007
*L. superbum*	[52]	KP462883	KP462883	KP462883	KP462883	KP462883
*L. bakerianum*	[53]	KY748301	KY748301	KY748301	KY748301	KY748301

**Table 3:** *Fritillaria* samples used in nrITS phylogenetic analyses together with GenBank accession numbers.

Taxa	References	ITS
*F. burnatii* (TEN1-20p2017)	this study	MT522874
*F. burnatii* (TEN2-04p2013)	this study	MT522877
*F. burnatii* (SER-05p2013)	this study	PV816804
*F. involucrata* (MEN-14p2016)	this study	MT522876
*F. meleagris* (Chase 2566 (K))	[11]	AY616730
*F. meleagris* (K:DNA:MWC12064)	[54]	HE656029
*F. moggridgei* (MAR-07p2014)	this study	PV816806
*F. moggridgei* (CRA-12p2014)	this study	MT522875
*F. moggridgei* (PLU-08p2014)	this study	PV816805
*F. tubaeformis* (GLE-10p2013)	this study	MT522878
*F. tubaeformis* (GLE-09p2015)	this study	MT522879
*L. superbum*	[55]	JF829386
*L. bakerianum* (G2013001)	[56]	KF851093

**Figure 1:**
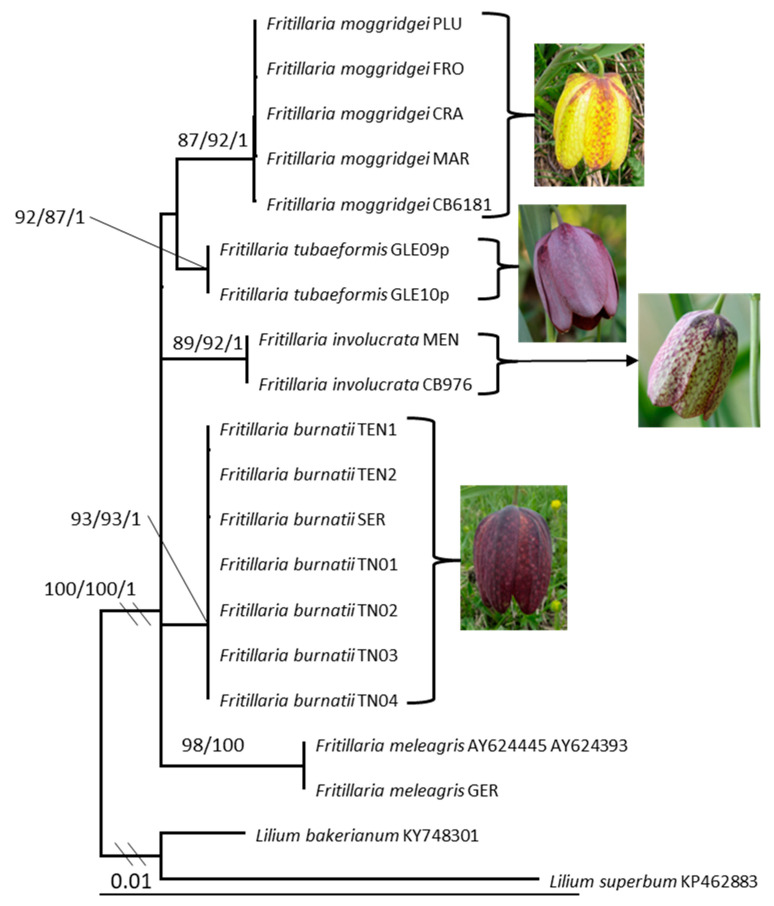
Maximum likelihood phylogram obtained from combined *mat*K, *ndh*F, *rpl*16, *rpo*C1, and *pet*A-*psb*J sequence alignment of *Fritillaria*. Values above or below branches indicate SH-aLRT support ≥ 80%/bootstrap support ≥ 70%/Bayesian posterior probabilities ≥ 0.95.

**Figure 2:**
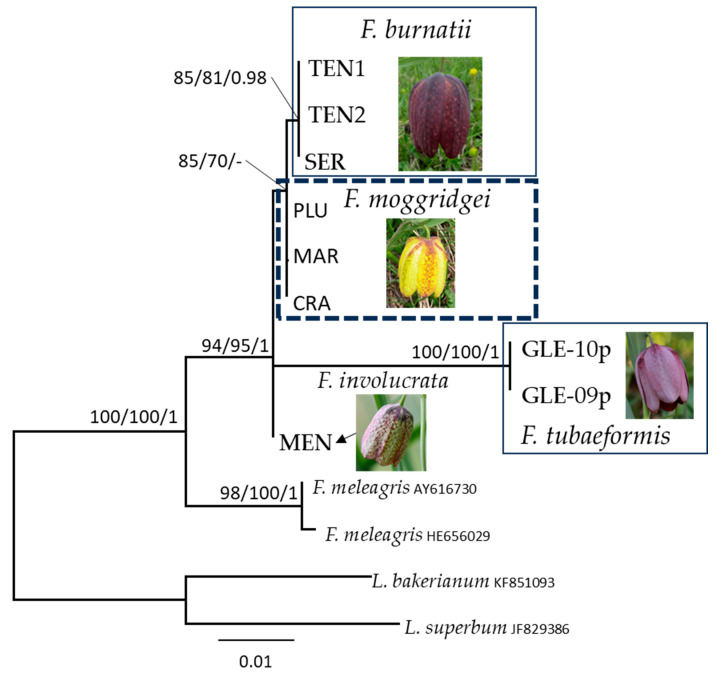
Maximum likelihood phylogram obtained from nrITS sequence alignment of *Fritillaria*. Only BPP values ≥ 0.90 and MLB values ≥ 70% are given above the clade branches. Values above or below branches indicate SH-aLRT support ≥ 80%/ML bootstrap support ≥ 70%/Bayesian posterior probabilities ≥ 0.95.

The SH-like approximate likelihood ratio test (with 1000 replicates) and bootstrap (1000 replicates) were used to evaluate the reliability of clades. Significant support is considered to be ≥80% for the SH-aLRT test, ≥70% for bootstrap support in the ML analysis (MLB), and ≥0.95 for BPP values in the Bayesian analysis (Figure 1 and Figure 2). The list of sequences used in our datasets are given in Table 2 and Table 3.

## 3. Results

### Molecular and Phylogenetic Analysis

Ten, fifteen, thirteen, eleven, and fourteen sequences were newly generated for *mat*K, *ndh*F, *rpl*16, *rpo*C1, *pet*A-*psb*J (Table 2), and nine sequences for nrITS (Table 3).

Both Bayesian and Maximum Likelihood analyses produced the same topology for both alignments and therefore, only the Maximum Likelihood tree with SH-aLRT test, MLB and BPP values is presented here (Figure 1 and Figure 2). The combined *mat*K*/ndh*F/*rpl*16*/rpo*C1*/pet*A-*psb*J data matrix comprises 20 terminal labels and contains 3105 total nucleotide sites. In the cpDNA phylogenetic analysis, the various taxa are consistently resolved into well-supported, distinct clades (see Figure 1). *Fritillaria burnatii*, *F. moggridgei*, *F. tubaeformis*, *F. involucrata*, and *F. meleagris* group in a highly supported clade (SH-aLRT = 100, MLB = 100 and BPP = 1; Figure 1). *F. moggridgei* from the Ligurian Alps, together with the accession from Maritime Alps, Tende (France) (Table 1) form a supported clade (SH-aLRT = 87, MLB = 92 and BPP = 1). All the accessions of *F. burnatii* from Ligurian and Maritime Alps (3), and from Trentino-Alto Adige (4) (Table 1) form a distinct lineage with a strong support (SH-aLRT = 93, MLB = 93 and BPP = 1; Figure 1). *F. burnatii* shows two characteristic deletions in *pe*tA-*psb*J intergenic spacer (TTCGG and AAAGAAT) and one deletion in *rpo*C1 intron region; the latter deletion is also shared with *F. involucrata* (GTAAA). Sequences of the French endemic *F. tubaeformis* form a supported clade (SH-aLRT = 92, MLB = 87 and BPP = 1; Figure 1) and show a 7 bp characteristic long indel (CTACATT) in the *rpl*16 intron.

The sequences of *F. involucrata* from the Ligurian and Maritime Alps (Table 1) group together in a highly supported clade (SH-aLRT = 89, MLB = 92 and BPP = 1) and show an exclusive insertion in the *rpo*C1 intron region (TTTTAGTT).

*F. meleagris* sequences grouped in a well-supported clade (SH-aLRT = 98, MLB = 100 and BPP = 1; Figure 1).

The nrITS alignment includes thirteen terminal labels and contains 622 sites. *Fritillaria burnatii*, *F. involucrata*, *F. moggridgei,* and *F. tubaeformis* form a well-supported clade (SH-aLRT = 94, MLB = 95 and BPP = 1; Figure 2) independent of *F. meleagris* interdigitated. Within this clade, *F. burnatii* is sister to *F. moggridgei*, but with weak support (SH-aLRT = 85, MLB = 70 and BPP = -), and they exhibit a sequence identity of 629/632 bp (99%) with 1 gap. The nrITS sequence identity between *F. burnatii* and *F. involucrata* is 469/475 bp (99%) with 1 gap. Within the alpine clade, *F. tubaeformis* diverges the most, showing an identity of 95% to 96% with the other species. In the Supplementary Materials (Figure S1a–e), five phylogenetic trees corresponding to the analysed plastid regions are presented to illustrate the capacity of each region to distinguish between different taxa. The central portion of the *matK* region used in the phylogenetic analysis (amplified with primers BF and CR, 446 bp) successfully distinguishes two species. The *rpl16* region separates three species, while the *ndh*F region effectively discriminates all five species under consideration. The *pet*A region distinguishes four species; however, the sample of *F. moggridgei* (FRO) is positioned ambiguously in the tree due to an incomplete sequence (210 bp out of a total of 691 bp). Finally, the *rpo*C1 region is capable of resolving all five *Fritillaria* species examined.

## 4. Discussion

In our study of the *Fritillaria tubaeformis* complex, previously investigated through a morphological approach [23], we aimed to refine species boundaries using molecular methods. Our results revealed that the alpine *Fritillaria* taxa represent distinct phylogenetic species, as supported by both chloroplast (cpDNA) and nuclear (nrITS) DNA analyses. The phylogenetic species concept—which emphasises evolutionary history and relies on genetic data to group individuals into monophyletic units—proved effective in achieving our research objectives. However, it is important to note that this approach to the species does not hold absolute value, as it remains a complex and widely debated framework in systematic biology.

In support of our results, all species analysed across the Alpine arc exhibit species-specific indels, which likely represent putative apomorphies and can be used as genetic markers for species circumscription. These indels, extensively documented in the results section, were not computed in our phylogenetic analyses and thus provide additional support for the recognition of *F. burnatii*, *F. involucrata*, *F. tubaeformis*, and *F. moggridgei* as distinct species.

**Figure 3:**
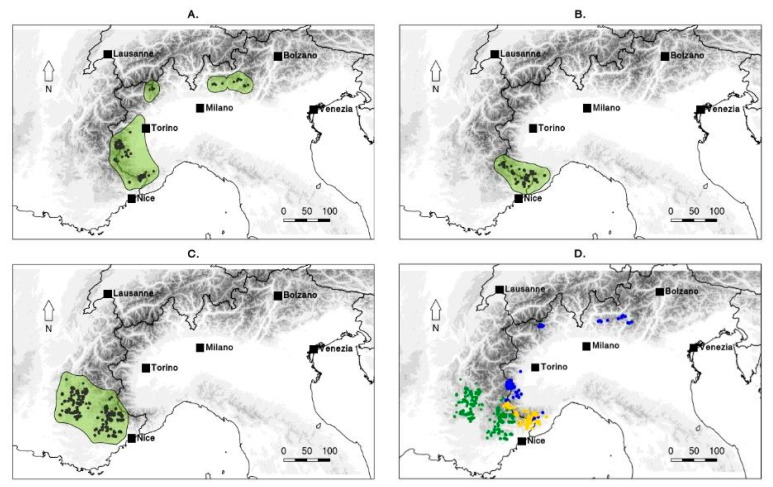
Geographical distributions of the three endemic taxa of *Fritillaria* based on available occurrence data. (**A**) *F. burnatii* (Planch.) Backh.; (**B**) *F. moggridgei* (Boiss. & Reut. ex Planch.) L.A. Cusin; (**C**) *F. tubaeformis* Gren. and Godr.; (**D**) Distributions of the three taxa have been overlaid in the map, representing population occurrences with dots of different colours: *F. burnatii* (blue dots), *F. moggridgei* (yellow dots), *F. tubaeformis* (green dots).

**Figure 4:**
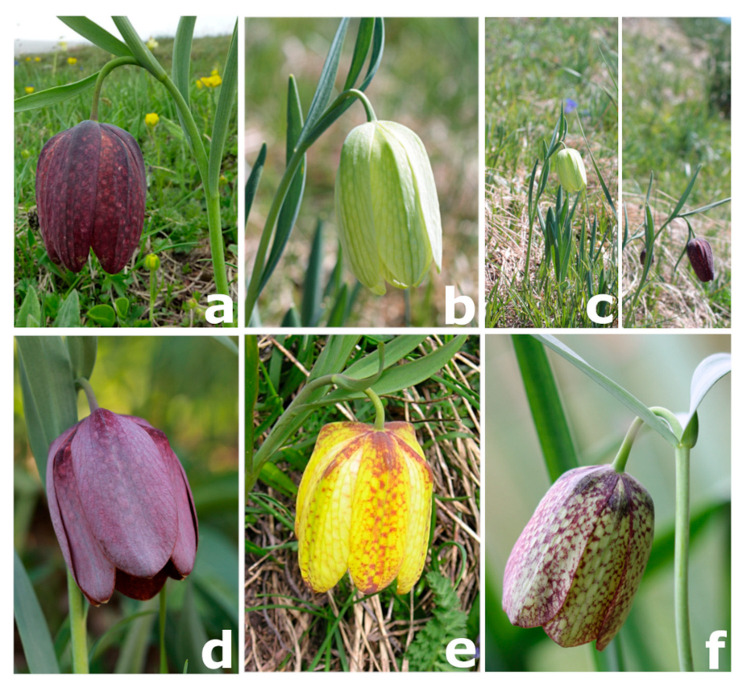
The *Fritillaria* taxa endemic of south-western Alps in their natural habitats: (**a**) *F. burnatii* (TEN1-20p2017); (**b**) *F. burnatii*, a close up of the individual bearing a discoloured perigone (TEN2-204p2013); (**c**) the same plant in the wild (left) and nearby individuals of *F. burnatii* with normal purple flowers (the image on the right has been cropped to put plants closer, same magnification; see text for details); (**d**) *F. tubaeformis* (GLE-10p2013); (**e**) *F. moggridgei* (FRO-00p2013); (**f**) *F. involucrata* (MEN-14p2016). In brackets, voucher codes of the sampled populations as in Table 1.

The plastid DNA analysis apparently supports a close relationship between *F. moggridgei* and *F. tubaeformis* but without statistical support, whereas the ML analysis of the nrITS region suggests a closer affinity between *F. moggridgei* and *F. burnatii*. This latter relationship, however, is not supported by the Bayesian analysis. Species delimitation is pivotal to understanding biological and biogeographical processes governing dynamics of plant communities and for the conservation of their population’s diversity. For the regions studied, no intraspecific variation was detected, except for the presence of some heterozygosity. In the nuclear nrITS sequences, different heterozygous sites were observed within sequences belonging to the same species. Particularly, our results supported the separation of *F. burnatii* (Planch.) Backh. from the Liguro-provençal microendemic *F. moggridgei* (Boiss. & Reut. ex Planch.) L.A.Cusin (Figure 3B) and from *F. tubaeformis* Gren. & Godr. The former has a range centred in Italy in the Ligurian and Maritime Alps, extending to Lombardy Prealps and Trentino (Figure 3A) [18,23,34]. Conversely, *F. tubaeformis* occurs throughout the French Préalpes, Haute-Alpes, Drôme, Basses-Alpes with a few populations on the Maritime Alps (Figure 3C). Until recently, *F. burnatii* and *F. tubaeformi*s have often been treated as the same species by several European authors, likely due to their similar purple-brown flowers (Figure 4a–d), and influenced by earlier classifications such as that proposed by Rix [29]. As already noticed, observation bias could have originated also from their close and, somehow, interdigitated (albeit never overlapping) distributions (Figure 3). However, in *F. burnatii*, the perigone is campanulate with a typical U profile. Tepals are sombre-purple or wine-purple with evident white or cream-yellow tessellation and they are shorter and narrower than in *F. tubaeformis* and in *F. moggridgei*. Differently, here perigones are large and with a sub-rectangular profile being flower elements angled at the point of inflexion. Moreover, tepals are uniformly purple, glaucous, and covered with pruina in *F. tubaeformis*; they are yellow ochre with a typical tessellation of purple lines, spots, and green veins in *F. moggridgei*.

Our phylogenetic analysis, based on four plastid regions (*ndh*F, *rpl*16, *rpo*C1, *pet*A-*psb*J) and the nuclear ITS region, support the morphological distinction within the *F. tubaeformis* group.

### 4.1. Fritillaria meleagris

*Fritillaria meleagris* shows a wide Eurasian distribution [57,58]. This species is native to south-eastern Europe with a scattered range extending to 52–53° N [58], from where it probably spread into north-western parts of Europe after forest grazing and clearance of woodlands. It was repeatedly considered as closely related to or identical with *F. burnatii*, also known as *F. meleagris* subsp. *burnatii* (Planch.) Rix [29]. However, *F. meleagris* is distinguished by the former for its nodding, bell-shaped flowers with a distinctive checkerboard pattern in shades of purple and white. Tepals are broad but markedly acute at the apex, and not pruinose, two traits rarely found in flowers of the *F. tubaeformis* complex. The occurrence of *F. meleagris* s.str. in French and Italian Alps was erroneously reported in the past [21,25] and even more recently [59]. Its occurrence in the Alps is limited to the easternmost border of the mountain chain [26], namely in Austria and Slovenia. In our nrITS phylogenetic analysis, *F. meleagris* was strongly supported as sister to the alpine *Fritillaria* included in this study (SH-aLRT = 100, MLB = 100 and BPP = 1; Figure 2), providing strong evidence that this is a well-circumscribed species.

### 4.2. Fritillaria burnatii

*Fritillaria burnatii* (Planch.) Backh. has its type locality in the Maritime Alps of Piedmont (Italy), at the very boundary with France, where it shows a well-defined range [18] (Figure 3A). In France, this taxon has remained long unknown and sometimes confused with *Fritillaria tubaeformis* s.str. Only more recently it has been recognised in the French floras [19,31]. Many Italian botanists have long misidentified this taxon with *F. meleagris* or *F. involucrata* [34], or even fully synonymised with *F. tubaeformis* (≡ *F. delphinensis*) [24,25,27,60]. When Bartolucci and Peruzzi [34] designated the lectotype based on a specimen collected by Planchon and conserved at G-BU, they argued its independent taxonomic status from *F. tubaeformis*, and provisionally maintained the validity of the taxonomic setting proposed by Rix [29], namely *Fritillaria meleagris* subsp. *burnatii* (Planch.) Rix. Based on morphometric data, Mucciarelli et al. [23] have clearly shown the separation between the two taxa. Specifically, it was shown that *F. burnatii* has narrower, markedly canaliculate and ± glaucous leaves, acute at apex and inward folding after drying. These in *F. tubaeformis* are only briefly canaliculate, the lower ones slightly obtuse, the upper ones acute at apex and never folding as in the previous species. In addition to differences in flower morphologies, previously highlighted in the text, we also observed significantly shorter ovaries and styles than in *F. tubaeformis* s.l. [23]. Therefore, also in accordance with the most recent floras of France [18] and the Italian checklist of vascular flora [21], all records of *F. tubaeformis* subsp. *tubaeformis* from Italy (ref. [28] have been referred to as *F. burnatii* [23,34,61]. Indeed, in this study, we have shown that *F. burnatii* and *F. tubaeformis* belong to independent lineages, confirming previous results [32]. In addition, *F. burnatii* is not closely related to *F. meleagris.* In our phylogenetic analysis, the accession collected in Valle Pesio (SER-05p2013), the two accessions collected in the type locality (Cima di Forte Pernante, Mont Piernaud, Cuneo), together with samples from Trentino clustered together in a well-supported clade (SH-aLRT = 93, MLB = 93 and BPP = 1; Figure 1). The analysis of the nrITS region also supports the placement of *F. burnatii* as an independent lineage (SH-aLRT = 85, MLB = 81, and BPP = 0.98; Figure 2), seemingly related to *F. moggridgei* but with low statistical support. The sample here labelled as TEN2-04p2013 corresponds to a single individual observed at Cima di Forte Pernante in spring 2013, occurring alongside typical *F. burnatii* individuals. This single individual showed a creamy yellow U-shaped perigone with pale greenish venations (Figure 4b,c), sensibly differing from the perigone of *F. moggridgei*, which is usually of a much darker yellow with green or purple tessellations (Figure 4e). Based on morphometric measurements made at Cima di Forte Pernante, discriminant analysis assigned this individual to *F. burnatii* with a 99% probability.

The occurrence of yellow or whitish perigones within *Fritillaria* populations usually showing purple flowers is already documented in the literature [62], and the possibility exists that albino variants occur in populations of these plants. Interestingly, the occurrence of a distinct “ivory whitish variant” of flower in *F. tubaeformis* s.str. (≡ *F. delphinensis*) was documented in 1800s [63] and also observed recently by one of us (VN).

### 4.3. Fritillaria tubaeformis and F. moggridgei

Our results demonstrated that the sampled populations of *Fritillaria moggridgei* are distinct from *F. tubaeformis*. The former has been treated, in the past, either as a subspecies, a variety or a full synonym of *F. tubaeformis* [34]. As mentioned before, *Fritillaria tubaeformis* shows uniformly dark purple perigones with no apparent tessellation (Figure 4d). In contrast, *F. moggridgei* shows yellow perigones with a green or purple tessellation (Figure 4e). Indeed, the shape of the perigone is very similar between the two taxa. This occurrence, combined with the fact that *F. moggridgei* occasionally co-occurs not with *F. tubaeformis* but rather with *F. burnatii* (yellow-blue dots, Figure 4d) increases the likelihood of misidentification, and may also erroneously suggest a genetic relatedness among the two taxa. The occasional occurrence in France of individuals of *F. burnatii* growing just a few metres from *F. moggridgei* has been reported by one of us (VN). In the past, the co-occurrence of *F. moggridgei* and *F. burnatii* in Italy was documented by Planchon [57], who, referring to a letter of Burnat, recorded a note of *F. burnatii* being interspersed to *F. moggridgei* at the boundary between the Pesio and Vermenagna Valleys (Colle del Carbone, Cuneo).

Based on the narrower outer tepals and longer outer nectaries, ovaries, stigma lobes and anthers, we were able to separate *F. moggridgei* from *F. tubaeformis* on morphological grounds [23]. However, acknowledging the general morphological overlap occurring between these two taxa, we firstly had deemed more opportune to recognise them at subspecies level, also considering that they show contiguous, partially interdigitated, but never overlapping ranges [18] (yellow-green dots, Figure 3D). Our results, however, point to a clear phylogenetic separation between the two taxa, whose sister relationship is not statistically supported. Genetic isolation by environment or ecology might have occurred between *F. tubaeformis* and *F. moggridgei*, as outlined by Dagnino et al. [64]. These authors found a relatively high degree of niche differentiation among the two taxa. Based on this observation, they inferred that *F. moggridgei* is a derivative taxon with a smaller range [64].

### 4.4. Fritillaria involucrata

*Fritillaria involucrata* has been included so far only in two large phylogenetic analyses at genus level [12,13] based on combined cpDNA datasets (*mat*K + *rbc*L + *rpl*16). Both papers agreed in placing *F. involucrata* (two accessions in Day et al. [12] and one accession in Huang et al. [13]) in a clade with *F. tubaeformis*. Our results do not support a close phylogenetic relationship of the two taxa and place *F. involucrata* in a strongly supported polytomy with the remaining alpine species (Figure 1 and Figure 2). The sequence of *F. tubaeformis* used by Day et al. [12] and by Huang et al. [13] correspond to a living accession at Kew (voucher Chase 2558) [11,12]. Images of the living specimen were not available at the time of publication [12,13,65] but it is still possible to determine the taxonomic identity of this material and distinguish it based on morphological traits. *F. involucrata*, like the taxa of the *F. tubaeformis* complex, has campanulate flowers. Yet, its tepals often display a distinctive mix of reddish-purple hues with yellow or greenish markings (Figure 4f). Both the background colour and tessellation pattern are highly variable in this species, a feature that may have caused some confusion or misidentification. However, a notable feature of *F. involucrata* is that the upper leaves are typically fused, forming a whorl of three leaves that resemble a false involucre.

## 5. Conclusions

Based on the morphological differences between the analysed samples outlined throughout the paper (see, for example, differences in morphology of tepals and leaves within the endemic species) along with the support received from phylogenetic analyses and the presence of characteristic indels, we believe that the specific status of these taxa was consistently confirmed. Despite the marked morphological variability, a clear congruence between morphological traits and phylogenetic species boundaries was demonstrated within the *F. tubaeformis* complex.

The scattered distribution pattern of these taxa has probably favoured the origin and maintenance of this diversity. Moreover, clonal propagation by bulbs, in addition to isolation in scattered refugial areas during the climatic oscillations of the Quaternary, might have contributed to the maintenance of relatively high levels of population differentiation as previously found [32,33]. Our phylogeny shed light on the taxonomic status of a group of *Fritillaria* species with a relatively large range in the Alps. Although the analysis does not fully resolve the relationships within the *Fritillaria tubaeformis* complex, it supports the phylogenetic independence of the three taxa, by providing additional insights on the high conservation value of the Alpine populations. Molecular evidence aligns well with key diagnostic traits, reinforcing the recognition of *F. tubaeformis*, *F. moggridgei*, and *F. burnatii* as three distinct species.

## Figures and Tables

**Table 1:** Sampled plant materials and voucher codes for the *Fritillaria* plastid regions.

Taxa	DNA Voucher Code (Herbarium Specimen) ^a^	Country	Site of Sampling	Locality
*Fritillaria burnatii*	TEN1-20p2017 (TO-HP 7481)	Italy	Maritime Alps	Colle di Tenda (Cuneo)
*Fritillaria burnatii*	TEN2-04p2013	Italy	Maritime Alps	Colle di Tenda (Cuneo)
*Fritillaria burnatii*	SER-05p2013 (TO-HP 7482)	Italy	Ligurian Alps	Vallone di Serpentera (Cuneo)
*Fritillaria burnatii*	TN01	Italy	Trentino-Alto Adige	Bondo–Breguzzo–Daone (Trento)
*Fritillaria burnatii*	TN02	Italy	Trentino-Alto Adige	Bondo–Breguzzo–Daone (Trento)
*Fritillaria burnatii*	TN03	Italy	Trentino-Alto Adige	Bondo–Breguzzo–Daone (Trento)
*Fritillaria burnatii*	TN04	Italy	Trentino-Alto Adige	Bondo–Breguzzo–Daone (Trento)
*Fritillaria involucrata*	MEN-14p2016 (TO-HG 3558)	Italy	Ligurian Alps	San Bernardo di Mendatica (Imperia)
*Fritillaria involucrata*	HYE-CB976	France	Maritime Alps	Saint-Vallier-de-Thiey (Grasse)
*Fritillaria meleagris*	GER-11p2015	Germany	Cultivated seedlings	Hortus Botanicus Taurinensis
*Fritillaria moggridgei*	MAR-07p2014 (TO-HP 7485)	Italy	Ligurian Alps	Vallone del Marguareis (Cuneo)
*Fritillaria moggridgei*	CRA-12p2014	Italy	Ligurian Alps	Vallone di Cravina (Cuneo)
*Fritillaria moggridgei*	FRO-00p2013	Italy	Ligurian Alps	Monte Frontè (Imperia)
*Fritillaria moggridgei*	PLU-08p2014 (TO-HP 7484)	Italy	Ligurian Alps	Pian del Lupo (Cuneo)
*Fritillaria moggridgei*	HYE-CB6181	France	Maritime Alps	Tende (France)
*Fritillaria tubaeformis*	GLE-10p2013	France	Hautes Alpes	Col de Gleize (Gap)
*Fritillaria tubaeformis*	GLE-09p2015 (TO-HG 3328)	France	Hautes Alpes	Col de Gleize (Gap)
*Lilium superbum*	Chase, M.W. 112	n/a	-	-
*Lilium bakerianum*	BOP040929	China	-	-

(^a^ n/a = not available; - = not applicable).

## Data Availability

Sequences employed in this study are openly available at www.ncbi.nlm.nih.gov/genbank/ (accessed on 25 June 2025). All herbarium specimens used in this study are kept in the collections of different institutions (see Table 1 for details).

## Supplementary Materials

The following supporting information can be downloaded at: https://www.mdpi.com/article/10.3390/biology14070785/s1, Figure S1a–e. Maximum Likelihood phylogenetic trees for each plastid region considered (a: *mat*K, b: *ndh*F, c: *rpl*16, d: *rpo*C1 and e: *pet*A-*psb*J).
