# Supplementary material for: Delimitation and Phylogeny in Fritillaria Species (Liliaceae) Endemic to Alps
Source: Biology (Basel). 2025 Jun 28;14(7):785. doi: 10.3390/biology14070785 (PMC12292719; doi:10.3390/biology14070785)
Supplement: Supplementary file 1 [file biology-14-00785-s001.zip › biology-3692938-supplementary.pdf]

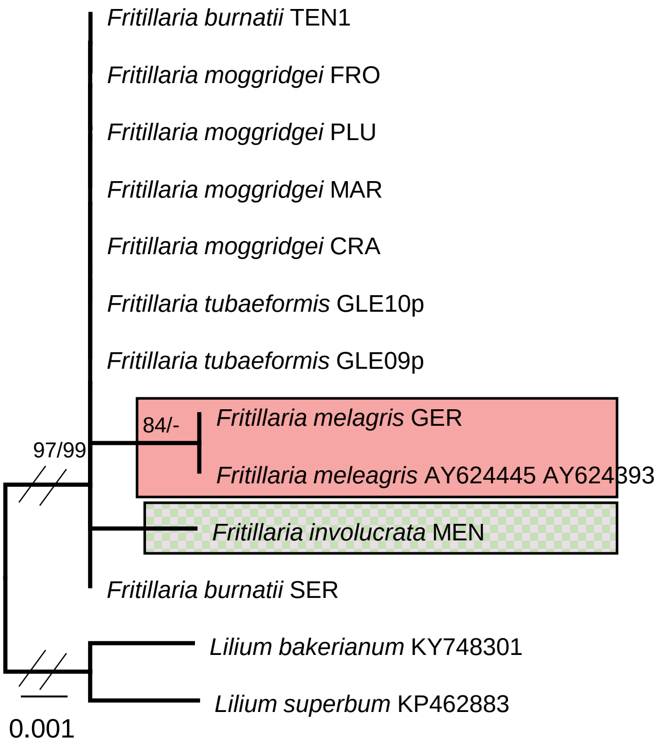

Figure S1a. Maximum likelihood phylogram obtained from *matK*. Values above branches indicate SH-aLRT support ≥ 80%/ ultrafast bootstrap support ≥ 95%.

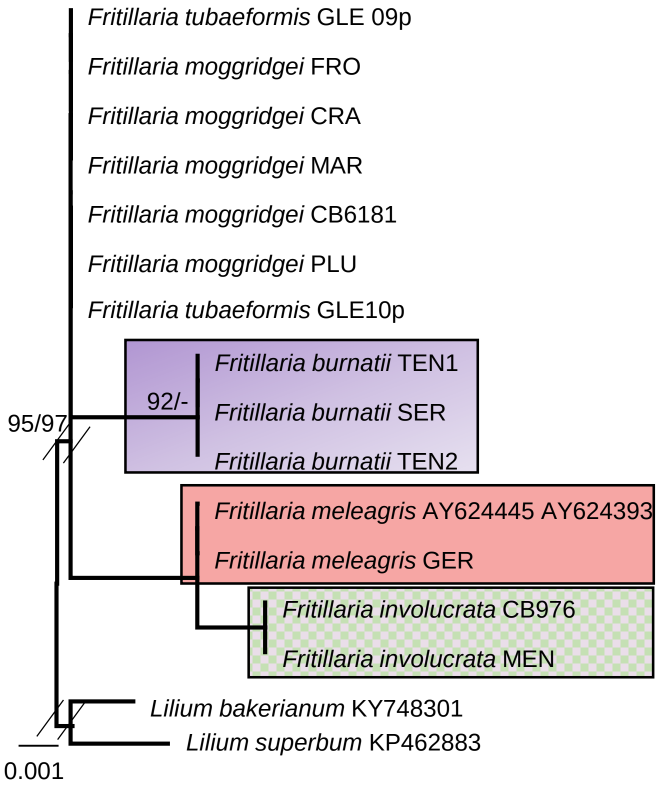

Figure S1b. Maximum likelihood phylogram obtained from *rpl16*. Values above branches indicate SH-aLRT support ≥ 80%/ ultrafast bootstrap support ≥ 95%.

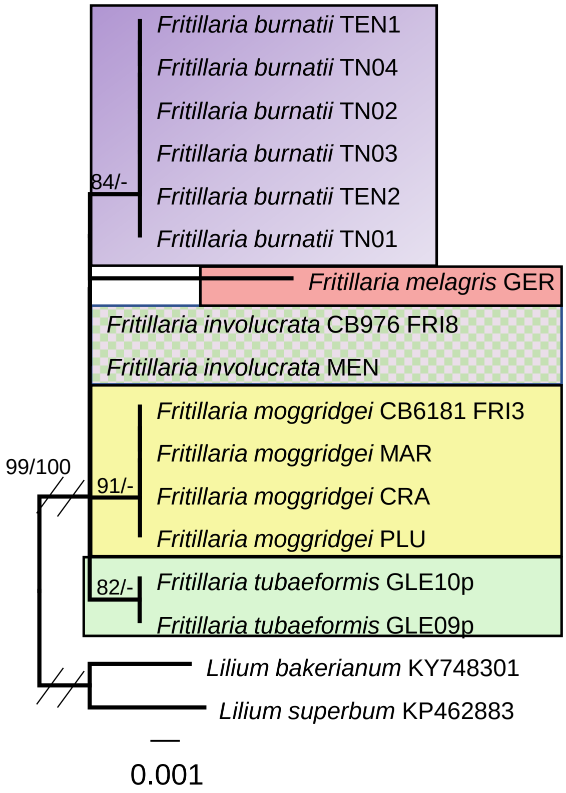

Figure S1c. Maximum likelihood phylogram obtained from *ndhF*. Values above branches indicate SH-aLRT support ≥ 80%/ ultrafast bootstrap support ≥ 95%.

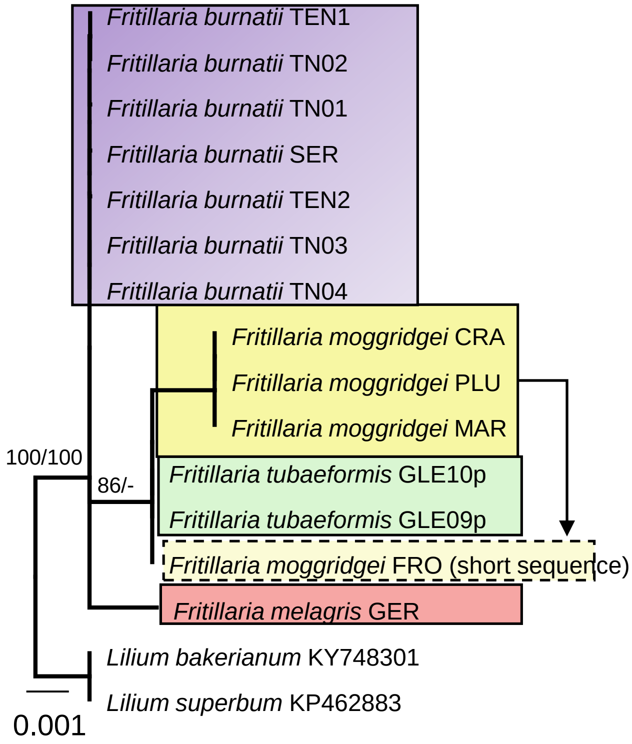

Figure S1d. Maximum likelihood phylogram obtained from *petA*. Values above branches indicate SH-aLRT support ≥ 80%/ ultrafast bootstrap support ≥ 95%.

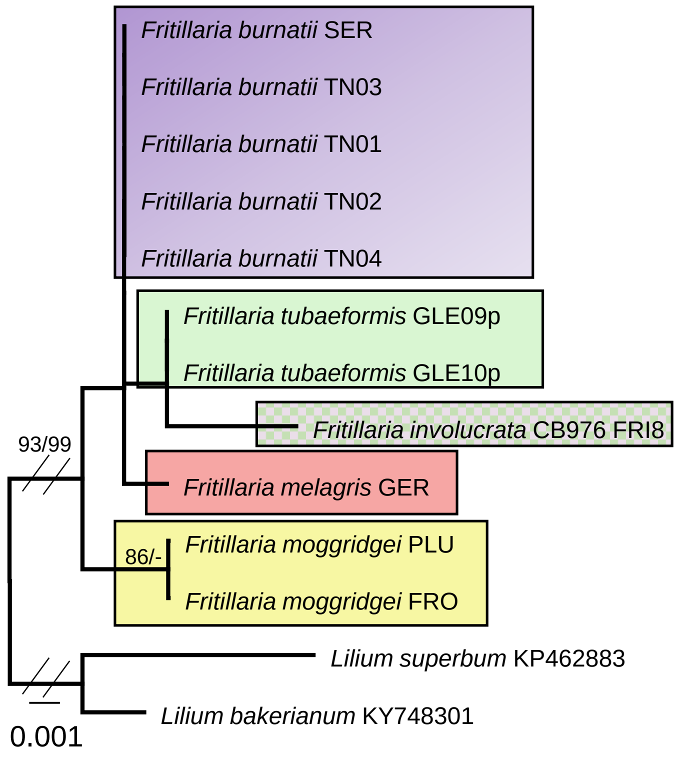

Figure S1e. Maximum likelihood phylogram obtained from *rpoC*. Values above branches indicate SH-aLRT support ≥ 80%/ ultrafast bootstrap support ≥ 95%.
